# One year of procarbazine lomustine and vincristine is poorly tolerated in low grade glioma: a real world experience in a national neuro-oncology centre

**DOI:** 10.1186/s12885-021-07809-5

**Published:** 2021-02-08

**Authors:** Rachel J. Keogh, Razia Aslam, Maeve A. Hennessy, Zac Coyne, Bryan T. Hennessy, Oscar S. Breathnach, Liam Grogan, Patrick G. Morris

**Affiliations:** 1 Department of Medical Oncology, Beaumont Hospital, Dublin 9, Ireland; 2 Cancer Clinical Trials and Research Unit, Beaumont Hospital, Dublin 9, Ireland; 3 Royal College of Surgeons Ireland, Dublin, Ireland

**Keywords:** Procarbazine, Lomustine, Vincristine, Chemotherapy, Glioma

## Abstract

**Background:**

Following optimal local therapy, adjuvant Procarbazine, Lomustine and Vincristine (PCV) improves overall survival (OS) in low-grade glioma (LGG). However, 1 year of PCV is associated with significant toxicities. In the pivotal RTOG 9802 randomised control trial, approximately half of the patients discontinued treatment after 6 months. As patients on clinical trials may be fitter, we aimed to further explore the tolerability of PCV chemotherapy in routine clinical practice.

**Methods:**

We conducted a retrospective study between 2014 and 2018 at a National Neuro-Oncology centre. Patients who had received PCV during this time period were included. The primary objective was to assess tolerability of treatment. Secondary objectives included evaluation of treatment delays, dose modifications and toxicities.

**Results:**

Overall, 41 patients were included, 24 (58%) were male and 21 (51%) aged ≥40 years. 38 (93%) underwent surgical resection and all patients received adjuvant radiotherapy prior to chemotherapy. The median number of cycles completed was 3,2,4 for procarbazine, lomustine and vincristine respectively. Only 4 (10%) completed all 6 cycles of PCV without dose modifications. There was a universal decline in dose intensity as cycles of chemotherapy progressed. Dose intensity for cycle 1 versus cycle 6 respectively: procarbazine (98% versus 46%), lomustine (94% versus 48%) and vincristine (93% versus 50%). Haematological toxicities were common. Six (14%) patients experienced Grade III-IV thrombocytopaenia and 13 (31%) experienced Grade III-IV neutropaenia.

**Conclusion:**

Toxicities are frequently observed with the PCV regimen in clinical practice. It might be preferable to adjust doses from the start of chemotherapy to improve tolerability or consider alternative chemotherapy, particularly in older patients with LGG.

## Background

Low grade gliomas (LGGs) account for 17 to 22% of primary brain tumours, diffusely infiltrate the central nervous system and cannot be cured by surgery alone [1]. Until recently, the World Health Organisation (WHO) classified LGGs based on histopathological features, and divided them into astrocytoma, oligodendroglioma and oligoastrocytoma, but this was updated in 2019 to include molecular tumour features [2]. Patients with LGG typically present at a younger age, with the peak incidence between 35 and 44 years of age and have longer overall survival (OS) than patients with high grade (III-IV) gliomas [3, 4]. Given the incurable but indolent nature of these tumours, they pose unique challenges for physicians in terms of treatment decisions and the management of tumour-related and treatment-related sequelae.

In fact, the optimal management of patients with LGG has been one of the most controversial areas in Neuro-Oncology. For some patients with newly diagnosed tumours, watchful waiting has been an accepted strategy [5]. Conversely, a high-risk group has been defined, including factors such as age > 40, astrocytoma histology, tumour diameter > 6 cm, tumour crossing the midline and the presence of neurological deficit prior to surgery [6]. Following the decision to operate, these prognostic variables have also been used historically to select patients for more intensive therapy such as radiotherapy and chemotherapy. One of the oldest combinations chemotherapy regimens is PCV, which consists of CCNU (lomustine), procarbazine and vincristine. Previous studies exploring the efficacy of this regimen have mainly focused on high grade tumours [7, 8]. For example, the Radiation Therapy Oncology Group (RTOG)-9402 and European Organisation for Research and Treatment of Cancer (EORTC) 26951 studies examined the use of PCV in addition to radiation (RT) compared to RT alone in patients with anaplastic oligodendroglioma (per histological diagnosis) [7, 8]. In both studies, a survival benefit was seen for the RT plus PCV combination arm in patients with 1p19q co-deleted tumours [7, 8].

Against this background the Radiation Therapy Oncology Group (RTOG) conducted the prospective randomised 9802 trial, which was initiated in 1998 and enrolled patient with high-risk LGG, defined as age > 40 years or with subtotal resection [9]. Patients were randomised to radiation alone (40 Gy in 30 fractions over 6 weeks) or radiation plus PCV chemotherapy every 8 weeks for 6 cycles (1 year). This study demonstrated a significant improvement with chemotherapy with a median OS of 13.3 years for patients treated with PCV compared to 7.8 years for the radiation alone group [4]. This magnitude of treatment benefit was considerable and established a new standard of care for LGGs. Despite a planned 6 cycles of treatment in this trial, the median number of cycles completed for procarbazine, lomustine and vincristine was three, four and four respectively [4]. The toxicities recorded were significant. The most clinically relevant of these being myelosuppression, most pronounced for neutrophils and platelets. Specifically, the incidence of grade III and IV haematological toxicity with PCV was 51 and 15% respectively [4].

It is unknown whether the broader use of the PCV combination in the real world would result in greater toxicity. Therefore, in our study, we aimed to gain a greater understanding of the tolerability of PCV in LGG in routine clinical practice.

## Methods

This was a retrospective study conducted in a national neuro-oncology centre in Ireland. Approval, including a waiver of informed consent was obtained per institutional guidelines. Patients with LGG who were treated with PCV chemotherapy between November 2014 and November 2018 were identified from a pharmacy database. Patient demographics and treatment data were collected from paper and electronic clinical records. As previously reported, the PCV regimen consisted of procarbazine (60 mg/m^2^ formulated in 50 mg capsules) orally day 8–21, lomustine (110 mg/m^2^ in 40 mg capsules) orally on day 1, and vincristine (1.4 mg/m^2^, capped at 2 mg) intravenously on day 8 and day 29 of each 56 day cycle [4]. Patients underwent dose reductions and delays as per standard of care.

The primary objective of the study was tolerability of treatment, as assessed by the number of patients completing all planned treatment without any dose omissions. Secondary objectives included treatment delays, dose modifications and the evaluation of toxicities graded as per CTCAE version 4 [10]. Data were collected for the overall population and using the age 40 as a cut-off as previously reported by Buckner et al. [4]. For the primary objective, the number of patients who had 1 or more drugs omitted was expressed as a percentage of the total number of patients for each cycle of PCV. Dose intensity was calculated for each chemotherapeutic agent by cycle as a percentage of the total calculated dose that was actually administered. Dose omissions, dose delays and reductions were recorded for each chemotherapy agent for each cycle of PCV. Data were collected using Microsoft Excel and statistical analysis was performed using Excel and SPSS™ version 26 software. Patient characteristics were reported as median for continuous variables, and frequencies and percentages were calculated for categorical variables. Baseline characteristics of those aged < 40 years were compared to those who were aged 40 and above were tested for dependence using chi-square test. Fisher’s exact was used when the expected frequencies proved to be too small.

## Results

### Patient characteristics

A total of 45 patients with LGG who received PCV chemotherapy were identified. Four patients were excluded: one had no prior radiotherapy, one had chemotherapy in another institution and two had additional radiotherapy in the preceding 5 years. Therefore, 41 patients were included in the study. Most (58%) patients were male (Table 1). There was a similar number of patients that were aged < 40 (*n* = 20) and ≥ 40 years (*n* = 21). The predominant histopathological subtype was oligodendroglioma (68%). The vast majority (93%) of patients underwent surgical resection (rather than biopsy alone) prior to chemotherapy and all patients underwent adjuvant radiotherapy prior to initiation of chemotherapy. The majority of tumours had some favourable biology, 93% had IDH mutation and 61% 1p19q co-deletion. Notable differences between those under 40 years old and those aged 40 and above include histological subtype and the presence of 1p19q codeletion (Table 1). The histological diagnosis of oligodrendroglioma was more common in those aged 40 and above (90% versus 45%, *p* = 0.002) and accordingly, 1p19 codeletion was therefore more likely to be present (86% versus 35%, *p* = 0.001).

**Table 1:** Patient Baseline Characteristics

	Age < 40 ***N*** = 20		Age ≥ 40 ***N*** = 21		Total ***N*** = 41 (%)		***p***-value
	N	%	N	%	N	%	
**Sex:**							0.412^a^
Male	13	65	11	52	24	58	
Female	7	35	10	48	17	41	
**Recurrent/First Diagnosis:**							0.427^a^
First Diagnosis	12	60	10	48	22	54	
Recurrent	8	40	11	52	19	46	
**Surgery:**							1.00^b^
Resection	18	90	20	95	38	93	
Biopsy	2	10	1	5	3	7	
**Histology:**							0.002^a^
Oligodendroglioma	9	45	19	90	28	68	
Astrocytoma	11	55	2	9	13	32	
**IDH:**							1.00^b^
Mutated	19	95	19	90	38	93	
Wildtype	0	0	0	0	0	0	
Unknown	1	5	2	9	3	7	
**1p19q Co-deletion:**							0.001^a^
Present	7	35	18	86	25	61	
Absent	10	50	2	9	12	29	
Unknown	3	15	1	5	4	10	
**MGMT**							
Methylated	8	40	6	29	14	34	
Unmethylated	1	5	0	0	1	2.4	
Unknown	11	55	15	71	26	63	
**ATRX**							
Mutated	5	25	1	5	6	15	
Wildtype	3	15	8	38	11	27	
Unknown	12	60	12	57	24	58	

Each value is represented by number of patients (N) and percentage of patients (%)

^a^Chi-square test for independence

^b^Fisher’s exact test

### Treatment delivered

**Fig. 1:**
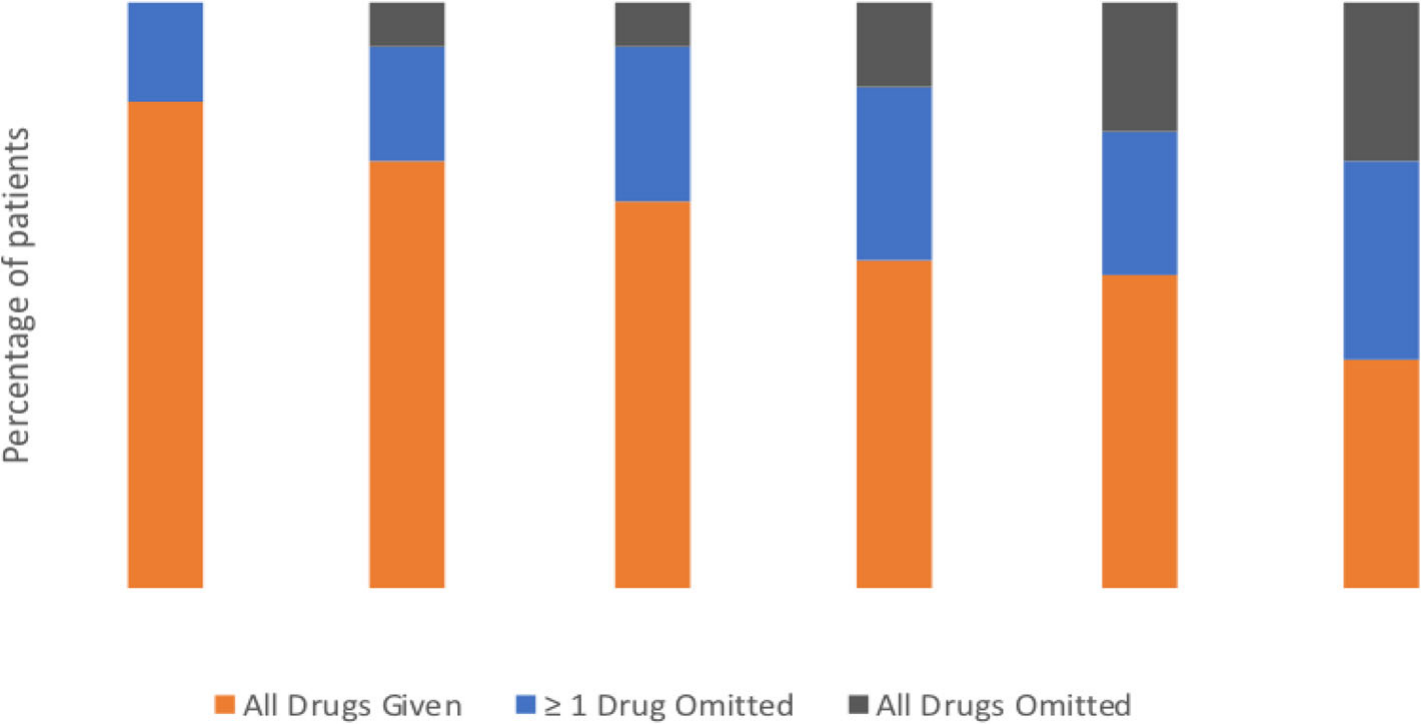
Treatment administered by PCV cycle. Data expressed as a percentage of patients who had all drugs given (orange), ≥1 ≥ 1drug omitted (blue), all drugs omitted (grey)

Twenty (48%) patients completed all 6 cycles of treatment. Only four (10%) patients completed all 6 cycles without any dose modifications. The median number of cycles completed of procarbazine, lomustine and vincristine was three, two and four respectively. The number of dose omissions increased as patients progressed from cycle 1 to cycle 6 (Fig. 1). In cycle 1, at least one dose was omitted in 17% of patients, but this rose to 29 and 34% in cycles 4 and 6 respectively. In addition, 15 and 27% patients had discontinued all treatment doses by cycle 4 and 6 respectively (Fig. 1).

**Fig. 2:**
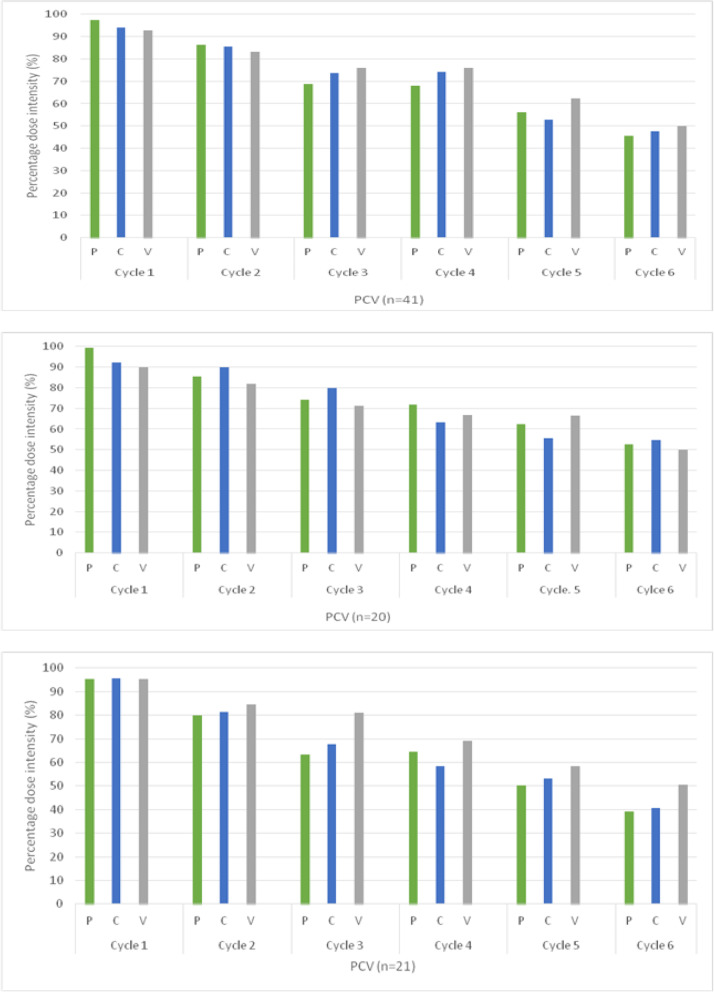
Dose intensity by cycle and by cytotoxic agent; **a**: Overall (*n* = 41), **b**: aged < 40 years (*n* = 20), **c**: aged ≥40 years old (*n* = 21)

The dose intensity for each chemotherapy agent decreased from cycle 1 to cycle 6 (Fig. 2a). The dose intensity for procarbazine dropped from 98% in cycle 1 to 68% in cycle 4 and 46% in cycle 6. Similarly, the dose intensity of lomustine dropped from 94% in cycle 1 to 74% in cycle 4 and 48% in cycle 6. For patients under the age of 40, the dose intensity for procarbazine, lomustine and vincristine was 98, 92 and 90% respectively in cycle 1. By cycle 6, the dose intensity for these three agents was 62, 56 and 67% respectively (Fig. 2b). This decline was even more evident in patients aged 40 years and above, for whom cycle 6 dose intensity was only 39, 41 and 50% for the three agents respectively (Fig. 2c).

**Fig. 3:**
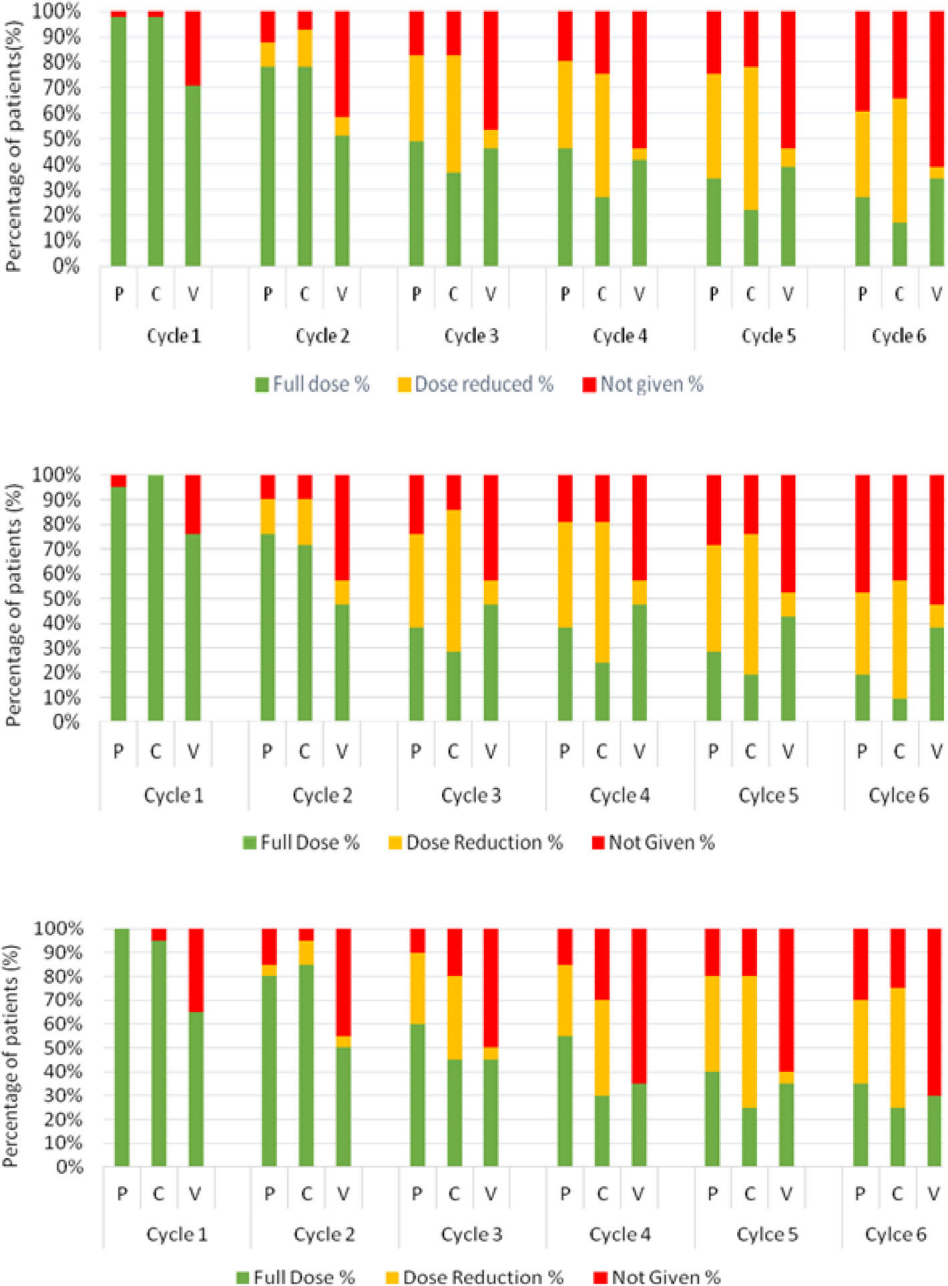
Percentage of patients who received full dose (green), dose reduction (yellow) and dose omission or delay (red) for each agent for each cycle of PCV. **a**: overall group (*n* = 41), **b**: < 40 years old (*n* = 20), **c** ≥40 years old (*n* = 21)

There was a consistent trend towards more dose reductions and omissions with increased cycles (Fig. 3). The percentage of patients who received the full dose of each drug for Cycle 1 as compared to Cycle 6 was as follows; procarbazine 98% versus 27%, lomustine 98% versus 17%, and vincristine 71% versus 34%. There were no dose reductions in Cycle 1 however by Cycle 6, 34% had a dose reduction of procarbazine, 49% had a dose reduction of lomustine and 5% had a dose reduction of vincristine. Only 2% of patients did not receive procarbazine or lomustine for Cycle 1, but this increased to 39 and 34% respectively for cycle 6. Overall, 60% of patients did not receive Vincristine for Cycle 6. Patients 40 and above received more dose reductions and dose omissions compared to those under 40 years of age for each of the three agents (Fig. 3b**/**c).

### Toxic effects

Overall, 21 (51%) patients had treatment discontinued prematurely. The most common reason for this was haematological toxicity, which occurred in 14 (66%) patients. In addition, 5 (24%) patients discontinued treatment due to non-haematological toxicity and two (9%) discontinued due to disease progression. In terms of haematological toxicities, 5 (12%) and 1 (2%) patients had Grade III and IV thrombocytopenia respectively. 10 (24%) and 3 (7%) patients had grade III and IV neutropaenia respectively. Table 2 summarises non-haematological toxicities recorded in our study most notably Grade I/II fatigue which occurred in 59% of cases.

**Table 2:** Most Common Toxicities

	Grade 1	Grade 2	Grade 3	Grade 4
**Haematological**				
Thrombocytopaenia	36	18	5	1
Neutropaenia	27	21	10	3
**Non-Haematological:**				
Fatigue	17	7	1	–
Gastrointestinal symptoms (anorexia, nausea)	15	7	1	–
Peripheral Neuropathy	11	–	2	–

Each value is represented by the number of patients that experience each toxicity (N)

## Discussion

This retrospective study conducted over a five-year period in a tertiary neuro-oncology centre provides important ‘real-world’ data on the tolerability of PCV chemotherapy in routine clinical practice. Our study confirms that frequent toxicities occur with the PCV regimen with implications for treatment tolerability. Less than half of the patients completed all 6 cycles and 10% of patients completed all 6 cycles without dose modifications, primarily because of haematological toxicity. Additionally, the number of dose reductions and dose delays increased as treatment progressed highlighting the associated toxicity and poor tolerability of this chemotherapy regimen. Perhaps unsurprisingly, PCV appeared to be less well tolerated in patients aged 40 and above, with a higher rate of dose reductions and dose delays seen in this cohort as compared to their younger counterparts, as well as a reduction in dose intensity. This likely reflects the better performance status of patients under the age of 40 and age is itself an independent risk factor for poor prognosis in LGG [6].

Patients with LGG have a longer survival than those with high grade tumours with a median survival of 13 years with intensive treatments [4]. Therefore, treatment toxicities are a critical consideration when determining the most appropriate therapeutic strategy. Given the relative longevity associated with these tumours, it is essential that treatment toxicities are carefully considered when making treatment decisions and managing tumour-related and treatment-related sequelae. In terms of non-haematological toxicities, we found that there was a higher incidence of Grade III nausea recorded in our study compared to the RTOG 9802 (5% versus 2%) [4]. However, Grade III fatigue was reported in 8% of cases in the RTOG 9802 trial compared 2% of cases in our study [4]. The RTOG 9802 had higher rates of Grade I/II non-haematological toxicities, which may reflect the fact that often less severe adverse events may be under-reported or not rigorously recorded in routine clinical practice. Although the incidence of peripheral neuropathy was not specifically recorded in the RTOG 9802 trial it should be noted that a high percentage of patients in our study experienced this (32%).

Our study found a higher rate of premature treatment discontinuation due to toxicity compared to the EORTC 26951 trial (46% versus 38%) [11]. Similar rates of grade III/grade IV neutropaenia and thrombocytopenia were reported when comparing our study to the EORTC 26951 trial (30% versus 32%, 17% versus 21%) [11]. In the RTOG-9402 trial, PCV was administered in an intensified regimen of 4 cycles of PCV every 6 weeks prior to RT, given at lomustine 130 mg/m^2^ orally on day 1; procarbazine 75 mg/m daily, days 8 through 21; and vincristine 1.4 mg/m^2^ intravenously on days 8 and 29 with no 2 mg limit on vincristine [12]. PCV was stopped prematurely in 20% of cases in the RTOG-9402 study due to toxicity [12]. In this dose-intense regimen, the rate of grade 3 and 4 toxicity was significant (33 and 32% respectively) with 80 of 144 patients in the PCV-RT arm experiencing severe haematological toxicity and two early deaths in the PCV-arm due to PCV-induced neutropaenia [7, 12]. Due to the adverse safety profile, the use of this dose-intense regimen is not seen in routine clinical practice. In the EORTC 26951 study, premature treatment discontinuation occurred in 38% of patients because of haematological toxicity (33%) or non-haematological toxicity (5%) [8].

The considerable number of dose reductions and dose omissions in our study, coupled with the fact that just over half of patients had treatment discontinued prematurely, supports the argument for adjusting doses from the outset of chemotherapy to improve tolerability. The impact of this on patient outcomes is unclear. Whether or not an alternative treatment strategy may be more suitable in patients with LGG remains to be seen. Temozolomide is easier to administer with better patient tolerance and it has improved survival in high grade glioma. It therefore has replaced PCV in more recent trials because of its improved toxicity profile and the expectation that both alkylating-based therapies would prove similarly efficacious. However, a direct comparison of the two regimens in this setting has yet to be completed. In the NOA-04 study, patients with newly diagnosed anaplastic gliomas were randomised to upfront RT or upfront chemotherapy (PCV or temozolomide) [13]. At progression, patients randomised to RT were treated with chemotherapy and those treated with upfront chemotherapy were treated with RT. Patients with 1p/19q-codeleted tumours treated with temozolomide upfront had a worse outcome compared to those treated with PCV regimen with a PFS of 4.46 years in the TMZ arm compared to 9.4 years in the PCV arm and OS of 8.09 years in the TMZ arm and not reached in PCV arm [13]. Primary chemotherapy (PCV or temozolomide) was inferior to upfront RT [13]. A potential hypothesis for the inferiority of temozolomide in the concern that temozolomide may lead to a hypermutatable phenotype. Johnson et al. performed genome sequence analysis of initial and recurrent gliomas with or without exposure to TMZ [14]. Patients with tumours previously treated with TMZ were hypermutated at recurrence in a greater proportion of TMZ-exposed patients [14]. The resulting genetic changes resembled those found in glioblastoma raising the possibility that TMZ could accelerate tumour transformation to a more aggressive phenotype. One of the limitations of this study is the lack of a PCV arm to compare the genetic alterations identified in recurrent tumours. The longest ongoing clinical trial comparing PCV versus TMZ in oligodendrogliomas is the Phase III CODEL trial which has reopened as a two-arm comparison of either RT followed by PCV or RT with concurrent and adjuvant temozolomide and the results of this are eagerly awaited [15]. This study includes both grade II and grade III oligodendrogliomas.

As more treatment options for low-grade gliomas become available, quality-of-life measures and outcomes will play key roles in management recommendations. Future therapies will be focused not only on improving survival but also on quality of life. As we move towards molecular profiling of tumours, more targeted and personalised treatments will hopefully be available and our treatment strategies will be altered accordingly.

Some of the limitations of this study should be noted. First, the sample size is relatively small, although this series still represents a large series given the rarity of the tumour. In addition to this, this is a retrospective study, which was unable to assess the impact of treatment on cognition and quality of life. Grade I and Grade II non-haematological toxicities may have been under-reported as we relied on retrospective clinical records for this information, rather that multiple additional data-points, which might have been collected in a clinical trial. Dose reductions may also not have been as rigorously or as consistently applied as in the RTOG 9802 trial. As such, data derived from this analysis should be interpreted cautiously.

In summary, our study confirms the earlier findings of the RTOG 9802 trial that the PCV regimen is poorly tolerated in routine clinical practice and demonstrates that toxicities are frequently observed. Furthermore, there was a notable difference in the treatment tolerability of PCV in those aged greater than 40. It might be preferable to adjust doses from the start of chemotherapy to improve tolerability or consider alternative chemotherapy, particularly in older patients with LGG.

## Data Availability

The data that support the findings of this study are available from the corresponding author on reasonable request.

## Abbreviations

PCV: Procarbazine, lomustine, vincristine
OS: Overall survival
RTOG: Radiation Therapy Oncology Group
LGG: Low grade glioma
WHO: World Health Organisation
EORTC: European Organisation for Research and Treatment of Cancer (EORTC)
CTCAE: Common Terminology Criteria for Adverse Events
RT: Radiation therapy
TMZ: Temozolomide
